# Dopaminergic Modulation of Glomerular Circuits in the Mouse Olfactory Bulb

**DOI:** 10.3389/fncel.2020.00172

**Published:** 2020-06-12

**Authors:** Shaolin Liu

**Affiliations:** Department of Anatomy, Howard University College of Medicine, Washington, DC, United States

**Keywords:** dopamine, synaptic transmission, interneuron, olfactory bulb, excitation, inhibition

## Abstract

Dopaminergic neurons are located in several brain areas including the olfactory bulb (OB) and involved in many physiological and pathophysiological processes. In the OB, dopamine (DA) is released exclusively by a population of interneurons termed short axon cells (SACs) in the glomerular layer, the initial synaptic integration site of the whole olfactory system. SACs corelease GABA and extend their processes to many glomeruli forming the interglomerular circuit. Two major groups of DA receptors D1-like (D1LRs) and D2-like (D2LRs) types are differentially distributed in the OB, i.e., D1LRs are broadly present except the most superficial olfactory nerve (ON) layer while D2LRs are predominantly confined to the ON and glomerular layers, suggesting that they mediate different physiological functions. In contrast to the well-known D2LR-mediated presynaptic inhibition of ON terminals in the OB, the cellular and circuit targets of the D1LR-mediated DA actions remain unclear even though D1LR activation improves odor detection and discrimination. We recently demonstrated that endogenous DA released from SACs or exogenous DA excites a population of excitatory glomerular neurons termed external tufted cells (ETCs) via D1LRs. But the physiological significance of this D1LR activation is largely unknown. In the present study, we addressed these questions by a systematic examination of exogenous DA actions on synaptic activities and excitabilities in most glomerular neurons and OB output neurons with the following major findings: (1) DA via D1LRs enhances OB output by potentiating the ETC-mediated feedforward excitation to the OB output neurons but suppresses spontaneous excitatory synaptic activities in both types of inhibitory glomerular interneurons periglomerular (PGCs) and SACs; (2) this suppression of excitatory synaptic activities in PGCs and SACs depends on activation of GABA_B_ receptors; (3) DA via D1LRs augments spontaneous inhibitory synaptic activities in all glomerular neurons and OB output neurons; (4) DA selectively activates SACs via D1LRs. These findings suggest that activation of D1LRs elevates the system’s sensitivity to odor stimuli and provide a mechanistic basis for the functional roles of DA in modulating odor detection and discrimination.

## Introduction

Dopamine (DA) as a neurotransmitter or neuromodulator plays important roles in many brain functions including motor control, motivation, reward, cognition, maternal and reproductive behaviors (Bjorklund and Dunnett, 2007a; Klein et al., 2019). DA actions are mediated by G protein-coupled receptors, which are classified into two major types based on their gene sequence homology and functions: the excitatory D1-like (D1LRs) and inhibitory D2-like (D2LRs) receptors (Beaulieu and Gainetdinov, 2011). In the mammalian brain DA-containing neurons are located in several distinctive cell groups distributed from the mesencephalon to the olfactory bulb (OB) (Dahlstroem and Fuxe, 1964; Bjorklund and Dunnett, 2007b).

In the OB, DA is exclusively released by a population of interneurons termed short axon cells (SACs), which express glutamic acid decarboxylase (GAD) 67, a key enzyme for biosynthesis of the inhibitory neurotransmitter GABA (Kosaka and Kosaka, 2008; Kiyokage et al., 2010). Both D1LRs and D2LRs are present in the OB with distinct distribution patterns, i.e., D1LRs are broadly present from glomerular to granule cell layers while D2LRs are confined only to the superficial olfactory nerve (ON) and the glomerular layers (Guthrie et al., 1991; Levey et al., 1993; Coronas et al., 1997; Gutierrez-Mecinas et al., 2005; Yu et al., 2019). Pharmacological activation of D2LRs (Nickell et al., 1991; Hsia et al., 1999; Ennis et al., 2001; Maher and Westbrook, 2008; McGann, 2013) or optogenetic activation of SACs inhibits glutamate release from ON terminals to postsynaptic cellular targets in the glomerular layer (Vaaga et al., 2017). Moreover, *in vivo* studies showed that blocking either D1LRs or D2LRs (Escanilla et al., 2009) or lack of DA transporters or D2LRs impairs odor discrimination in animals (Tillerson et al., 2006; Taylor et al., 2009) whereas olfactory deprivation upregulates D2LR density in the OB (Guthrie et al., 1991). Interestingly, a mating-triggered DA surge in the main OB of female mice impairs their perception of social odors contained in male urine (Serguera et al., 2008). Additionally, dopaminergic SACs exhibit high level of activity-dependent plasticity. For example, naris closure leads to a drastic reduction in the number of TH-immunoreactive cells and processes (Cave and Baker, 2009; Parrish-Aungst et al., 2011; Lazarini et al., 2014; Bonzano et al., 2016). Furthermore, majority of DAergic SACs originate from postnatal neurogenesis and are continuously subject to replenishment during adult life (Lazarini et al., 2014; Bonzano et al., 2016). All these previous findings suggest that DA actively participates in modulating signal processing in the OB. However, the mechanistic actions of DA at the cellular and circuit levels in the OB are not well understood.

The DAergic/GABAergic SACs have somata and processes confined to the glomerular layer (Kosaka and Kosaka, 2008; Kiyokage et al., 2010), where excitatory and inhibitory circuits are formed among the axonal terminals of olfactory sensory neurons (OSNs), apical dendrites of ETCs and the OB output neurons mitral and tufted cells (MTCs), and two major populations of inhibitory interneurons types – SACs and periglomerular cells (PGCs) (Lledo et al., 2005; Wilson and Mainen, 2006; Nagayama et al., 2014; Burton, 2017; Pignatelli and Belluzzi, 2017). Thus, the DAergic/GABAergic SACs are well-situated to modulate olfactory signal processing at the initial site of synaptic integration in the whole olfactory system. Physiological evidence suggests that functional operation of the SAC-derived circuit depends on postsynaptic cellular targets. For instance, DA is coreleased with GABA from SACs to mediate a biphasic excitatory-inhibitory sequential response in ETCs (Liu et al., 2013) while the transmission from SACs to MTCs or PGCs is mediated by GABAergic and electrical synapses (Banerjee et al., 2015; Liu et al., 2016) or GABA alone but not by DA (Shao et al., 2019). Alternatively, DA potentially functions as a neuromodulator in the OB through volume transmission (Cragg et al., 2001). A recent study revealed that DA release from SACs in the OB was quantal and calcium-dependent but was asynchronous and lasted for tens of seconds (Borisovska et al., 2013), supporting volume transmission. Thus, in the present study we designed experiments to examine actions of exogenous DA on both excitatory and inhibitory transmission in the glomerular circuit and analyzed the underlying cellular mechanisms.

## Materials and Methods

### Animals

Both male and female C57BL/6J mice were purchased from Charles River. The GAD2gfp mice provided of courtesy by Dr. Gabor Szabo were initially from the GAD65_3e/gfp5.5 #30 line on a genetic background of C57BL6 backcrossed to B6CBAF1/J wild-type mice to generate the transgene heterozygotes (Lopez-Bendito et al., 2004). TH-GFP mice were initially courtesy of Dr. Kobayashi (Matsushita et al., 2002) and generated using a 9.0 kb 5′-flanking region of the TH gene on a genetic background of C57BL/6J to crossbreed with DBA/2J mice. These TH-GFP transgenic mice were then backcrossed to C57BL/6J mice to generate heterozygotes for experimental usage. All animals were maintained with a standard 12-h light/dark cycle with *ad libitum* access to food and water. All experimental procedures were carried out in accordance with protocols submitted to and approved by the Howard University Institutional Animal Care and Use Committee.

### Slice Preparation

Acute OB slices were prepared from 6- to 8-week-old male or female mice as described previously (Liu et al., 2013). Briefly, horizontal slices (350 μm) were cut with a VT1200s vibratome (Nussloch, Germany) in an ice-cold and oxygenated (95% O_2_–5% CO_2_) sucrose-based artificial CSF (sucrose-ACSF) containing (in mM) 210 sucrose, 3 KCl, 1.2 NaH_2_PO_4_, 2.6 MgSO_4_, 0.5 CaCl_2_, 26 NaHCO_3_, 10 glucose. After 30 min incubation in normal ACSF at 30°C, slices were then transferred to ACSF at room temperature until they were used for recordings. Normal ACSF was continuously bubbled with 95% O_2_–5% CO_2_ and had the following composition (in mM): 124 NaCl, 3 KCl, 1.25 NaH_2_PO_4_, 2.0 MgSO_4_, 2.0 CaCl_2_, 26 NaHCO_3_, 10 glucose. During experiments, slices were perfused at 3 ml/min with ACSF equilibrated with 95% O_2_–5% CO_2_ and warmed to 30°C.

### Electrophysiology

Whole cell patch clamp recordings were made from OB neurons visualized using Axio Examiner (Zeiss, Oberkochen, Germany) fixed-stage upright microscope with near-infrared differential interference contrast (DIC) optics. Short axon (SACs) and periglomerular cells (PGCs) were identified by their expression of TH- or GAD65-EGFP in corresponding transgenic mice, respectively.

External tufted cells (ETCs) were initially reported in previous studies with Golgi staining (Romon and Cajal, 1909; Pinching and Powell, 1971a, b; Macrides and Schneider, 1982). Recent work more rigorously characterized the morphological and electrophysiological properties of ETCs (Hayar et al., 2004b; Antal et al., 2006). Based on findings from these studies, we redefined and identified ETCs with the following criteria: (1) spontaneous and intrinsic burst spiking that is resistant to blockers of fast synaptic transmitter receptors; (2) soma residing in the deep half of the glomerular layer with “pear” appearance viewed in near-IR DIC optics; (3) an apical dendrite tuft extensively ramifying in a single glomerulus but lack of lateral dendrites in the external plexiform layer (EPL).

To localize apical dendrite tufts of the recorded mitral/tufted cells (MTCs) in the glomerular layer, Alexa-594 (10 μM) was added to the recording electrode solution. Whole cell current or voltage signals were recorded with a MultiClamp 700B amplifier (Molecular Devices, Palo Alto, CA, United States) and low-pass filtered at 4 kHz and sampled at 10 kHz with a DIGIDATA 1550B 16-bit analog-to-digital converter (Molecular Devices) using Clampex 11.0.3 (Molecular Devices). Patch recording electrodes were pulled from standard-wall glass capillary tubes without filament (Sutter Instrument, Novato, CA, United States). Patch pipettes (4–7 MΩ) for whole cell current clamp recording contained (in mM) 115 K-gluconate, 5.0 EGTA, 0.63 CaCl_2_, 5.5 MgCl_2_, 10 HEPES, 3 Na_2_-ATP, 0.3 Na_3_-GTP, and 14 Tris-phosphocreatine (pH 7.3, 285–295 mOsm). Voltage clamp recordings of spontaneous excitatory (sEPSCs) or inhibitory postsynaptic currents (sIPSCs) were made with internal solution containing (in mM) 133 CsCH_3_O_3_S, 3 EGTA, 0.4 CaCl_2_, 5 QX-314, 4 MgCl_2_, 10 HEPES, 3 Na_2_-ATP, 0.3 Na_3_-GTP (pH 7.3, 285–295 mOsm). Cells were voltage clamped at −70 mV, the reversal potential of Cl^–^, to minimize the Cl-mediated sIPSCs thus to optimize the recording of sEPSCs. Cells were voltage clamped at 0 mV, the reversal potential of AMPA and NMDA receptor-mediated sEPSCs, to optimize recording of sIPSCs. Before bath application of the GABA_A_ receptor blocker gabazine (GBZ), NBQX (10 μM) and APV (50 μM) were pre-applied to block fast glutamate receptors thus preventing epileptic activity.

### Electrical Stimulation

Electrical stimulation was delivered by bipolar glass electrodes made from theta borosilicate tubes (Sutter Instrument). The isolated and constant current stimulation pulses (100 μs) were triggered by a Master-9 stimulator (AMPI, Jerusalem, Israel).

### Data Analysis

Amplitudes and frequencies of sEPSCs or sIPSCs were measured with Wdetecta.^1^ Drug effects on sEPSCs and sIPSCs were determined by measuring these parameters from 1 min duration traces taken immediately before or 1 min (micropuffing) or 2 min (bath application) after drug application in each condition for each cell. Other data were measured and analyzed with Clampfit 11.0.3 (Molecular Devices) and Origin Pro 2019 (Origin Lab, Northampton, MA, United States). Statistical significance of population responses was calculated by using paired Student’s *t*-test or ANOVA One-way repeated measure with Bonferroni *post hoc* comparisons in Origin Pro 2019.

### Drugs Delivery and Chemicals

Drugs were applied by either bath perfusion to treat the whole slices or micropuffing to target the relevant glomeruli by 2 ms and 30 psi pneumatic pressure generated by a picospritzer (Parker Instruments, Cleveland, OH, United States). Micropuffing pipettes were made from thick wall borosilicate glass capillaries without filament (Sutter Instrument) with a 6-μm tip diameter. These parameters were calibrated to deliver an injection volume of ∼20 nl and were previously shown to deliver drug coverage restricted to a single glomerulus (Shao et al., 2012).

D-2-Amino-5-phosphonopentanoic acid sodium salt (APV, 50 μM), 2,3-Dioxo-6-nitro-1,2,3,4-tetrahydrobenzo[f]quinoxaline-7-sulfonamide disodium salt (NBQX disodium salt, 10 μM), Gabazine (SR95531, 10 μM), 8-Bromo-2,3,4,5-tetrahydro-3-methyl-5-phenyl-1H-3-benzazepin-7-ol hydrobromide (SKF83566 bromide, 10 μM), (S)-(-)-5-Aminosulfonyl-*N*-[(1-ethyl-2-pyrrolidinyl)methyl]-2-methoxybenzamide [(S)-(-)-Sulpiride, 100 μM], (2S)-3-[[(1S)-1-(3,4-Dichlorophenyl)ethyl]amino-2-hydroxypropyl](phenylmethyl)phosphinic acid hydrochloride (CGP55845 hydrochloride, 10 μM) were purchased from Tocris Cookson (Ellisville, MO, United States). Dopamine chloride (DA, 20 μM or 100 μM) and all other chemicals were purchased from Sigma-Aldrich (St. Louis, MO, United States). All drugs were dissolved in distilled water as stock solution and diluted 1000 times with ACSF to final concentrations.

## Results

### Dopamine Inhibits Spontaneous EPSCs in Glomerular Interneurons via D1LRs

**FIGURE 1:**
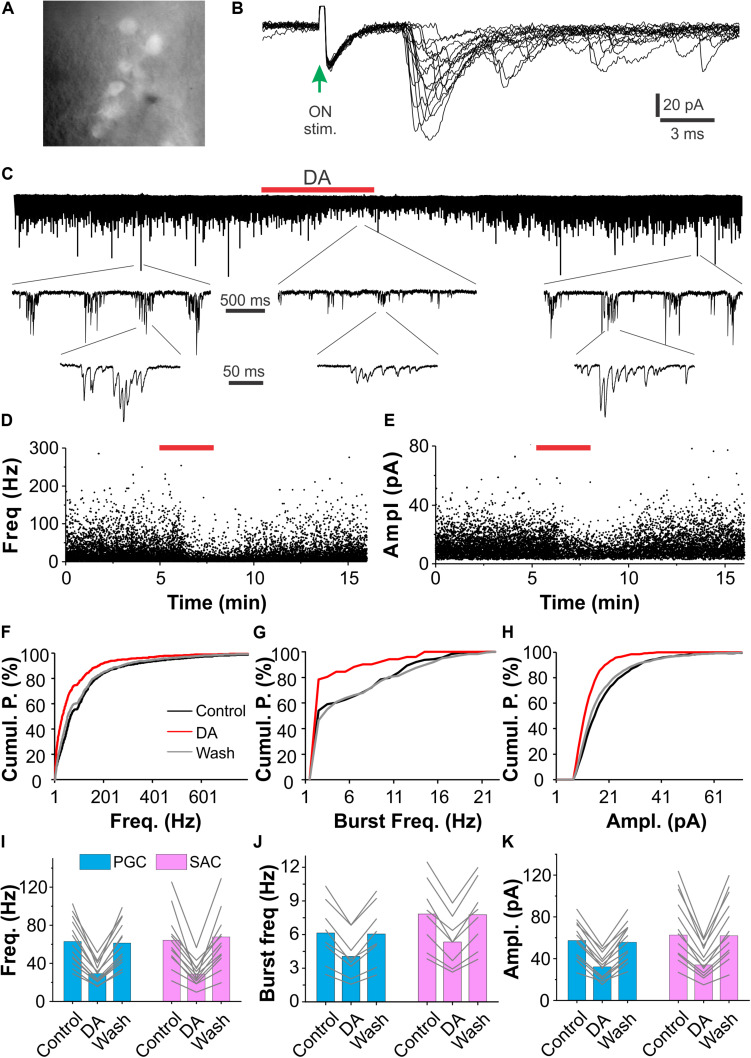
DA suppresses sEPSCs in glomerular interneurons. **(A)** Photo graph showing GFP-expressing periglomerular cells (PGCs) in the glomerular layer of an OB slice prepared from a GAD65-GFP mouse. **(B)** Typical excitatory postsynaptic current (EPSCs) traces in a PGC evoked by stimulation of the olfactory nerves (ON-stim.). Note that EPSC onset latencies are longer than 3 ms and varies among traces. **(C)** A representative voltage clamp recording trace showing that bath-applied DA (20 μM, red horizontal bar) reduces both frequency and amplitude of spontaneous burst EPSCs in a PGC (top). Middle and bottom traces are blown-up. **(D,E)**: plotting graphs showing changes in instant frequency **(D)** and amplitude **(E)** of sEPSCs before, during and after DA application in the same trace in **(C)**. **(F–H)** Line graphs of cumulative probability (Cumul. P.) against frequency of individual **(F)** or burst **(G)** sEPSCs, or amplitude (Ampl., **H**) of individual sEPSCs before (control), during (DA), and after (wash) DA application. **(I–K)** Pooled data showing that DA reduces both frequency of individual **(I)** or burst **(J)** sEPSCs and amplitude **(K)** of sEPSCs in both PGCs (*n* = 13) and short-axon cells (SACs, *n* = 12).

Previous studies have shown that optogenetic activation of SACs leads to GABA and DA corelease (Liu et al., 2013). The coreleased GABA and DA orchestrate to modulate ETC physiological behaviors in such a way that DA enhances *I*_h_ to boost the rebound excitation following the GABA_A_ receptor-mediated inhibition (Liu et al., 2013). Consistently, exogenous DA increases the *I*_h_-dependent bursts of action potentials in ETCs. As a major excitatory element in the glomerular circuit, ETCs monosynaptically drive vast majority of the inhibitory periglomerular cells (PGCs) and SACs (Shao et al., 2009; Kiyokage et al., 2010). All these findings lead us to predict that DA increases spontaneous excitatory synaptic activities in the ETC-driven PGCs and SACs. To test this, we recorded sEPSCs in PGCs and SACs in OB slices under voltage clamp and examined effects of exogenous DA. As PGCs and SACs exclusively express the key enzyme of GABA biosynthesis GAD65 and the rate-limiting enzyme of dopamine biosynthesis tyrosine hydroxylase (TH), respectively (Kiyokage et al., 2010), PGCs and SACs were accordingly identified in GAD65-GFP (Figure 1A) and TH-GFP (not shown) mice. To differentiate the ON-driven and ETC-driven PGCs and SACs, we started with recording their responses to ON stimulation. Consistent with previous findings (Shao et al., 2009; Kiyokage et al., 2010), vast majority of PGCs (8/13) or SACs (8/12) respond to ON-stimulation with long and inconsistent latencies (4.3 ± 0.31 ms, *n* = 8 PGCs; 4.6 ± 0.42, *n* = 8 SACs) EPSCs and exhibit bursts of sEPSCs (Figures 1B,C), indicating that they are ETC-driven glomerular interneurons. The rest PGCs (5/13) and SACs (4/12) responded to ON stimulation with short latencies (2.3 ± 0.15 ms, *n* = 5 PGCs; 2.1 ± 0.17, *n* = 4 SACs), suggesting that they are ON-driven glomerular interneurons. Surprisingly, bath application of DA reversibly suppressed sEPSCs in all tested PGCs (*n* = 13, Figures 1C–F,H) and SACs (*n* = 12). sEPSC frequency and amplitude in PGCs (*n* = 13) were 63.0 ± 6.7 Hz and 57.4 ± 5.0 pA in ACSF, 29.2 ± 3.0 Hz (*P* < 0.0001 vs. in ACSF) and 32.3 ± 3.4 pA (*P* < 0.0001 vs. in ACSF) in DA (20 μM), and 61.3 ± 6.3 Hz (*P* = 1 vs. in ACSF) and 55.7 ± 5.0 pA (*P* = 1 vs. in ACSF) after DA washout. Similarly, sEPSC frequency and amplitude in 12 SACs were 64.3 ± 8.4 Hz and 71.0 ± 9.0 pA in ACSF, 28.8 ± 3.6 (*P* < 0.0001 vs. ACSF) and 37.4 ± 4.3 pA Hz (*P* < 0.0001 vs. ACSF) in DA, 63.6 ± 8.5 Hz (*P* = 1 vs. ACSF) and 70.3 ± 8.8 pA (*P* = 1 vs. ACSF) after DA washout (Figures 1I,K). While the DA suppression of sEPSCs in the ON-driven PGCs and SACs is consistent with previous findings that DA inhibits glutamate release from the ON terminals (Hsia et al., 1999; Berkowicz and Trombley, 2000; Ennis et al., 2001; Maher and Westbrook, 2008; Vaaga et al., 2017), the similar DA action on the ETC-driven PGCs and SACs may indicate dopaminergic modulation of glutamate release from ETC apical dendritic terminals in the glomerular layer. Given that burst sEPSCs reflect spontaneous burst firing activities in presynaptic ETCs, we also measured the sEPSC burst frequencies and found that the frequency of sEPSC bursts was also reduced by DA (Figures 1G,J). sEPSC burst frequencies in PGCs (*n* = 8) and SACs (*n* = 8) were 6.1 ± 1.0 Hz and 7.8 ± 1.1 Hz in ACSF, 4.1 ± 0.7 Hz (*P* < 0.0001 vs. ACSF) and 5.3 ± 0.8 Hz (*P* < 0.0001 vs. ACSF) in DA, 6.1 ± 0.9 Hz (*P* = 1 vs. ACSF) and 7.7 ± 1.0 Hz (*P* = 1 vs. ACSF) after DA washout, respectively. Collectively, these results indicate that DA inhibits glutamate release from presynaptic ETC apical dendrites to PGCs and SACs thus reduces burst sEPSCs.

**FIGURE 2:**
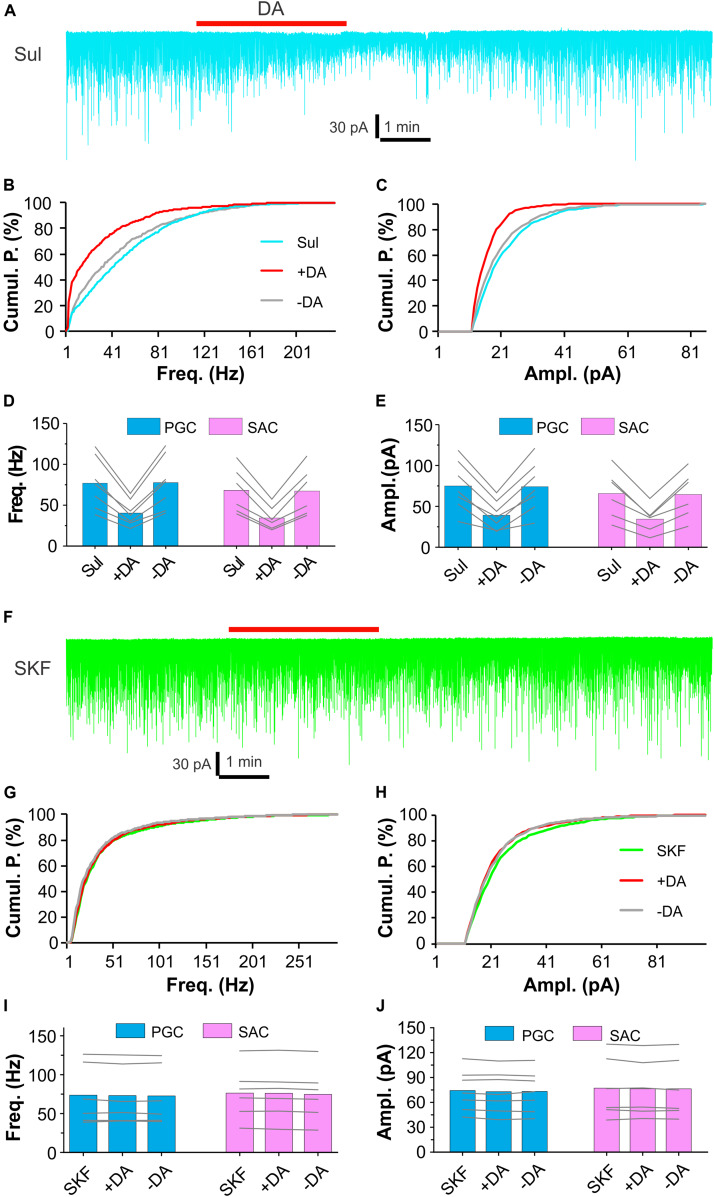
D1LRs mediate DA actions on sEPSCs in glomerular interneurons. **(A)** A representative recording trace showing DA effects on sEPSCs in a PGC in the presence of 100 μM (S)-(-)-Sulpiride (Sul), a selective D2LR antagonist. **(B,C)** Graphs plotting cumulative probability against frequency **(B)** or amplitude **(C)** of sEPSCs shown in **(A)**. **(D,E)** Pooled data showing that DA reduces both frequency **(D)** and amplitude **(E)** of sEPSCs in both PGCs (*n* = 7) and SACs (*n* = 6) in the presence of (S)-(-)-Sulpiride. **(F)** A representative recording trace showing that DA has no effect on sEPSCs in a PGC in the presence of 10 μM SKF83566 (SKF), a selective D1LR antagonist. **(G,H)** Graphs plotting cumulative probability against frequency **(G)** or amplitude **(H)** of sEPSCs shown in **(F)**. **(I,J)** Pooled data showing that DA has effect on neither frequency **(I)** nor amplitude **(J)** of sEPSCs in either PGCs (*n* = 7) or SACs (*n* = 6) in the presence of SKF83566.

Dopamine actions are mediated by two major categories of G protein-coupled receptors – D1LRs and D2LRs (Beaulieu and Gainetdinov, 2011). Evidence shows that D2LRs in ON terminals mediate presynaptic inhibition of glutamate release to their postsynaptic targets in the glomerular layer (Nickell et al., 1991; Aroniadou-Anderjaska et al., 2000; McGann, 2013). This explains why DA suppresses sEPSCs in the ON-driven PGCs and SACs. All ETCs receive direct ON input (Hayar et al., 2004a; De Saint et al., 2009; Gire and Schoppa, 2009), does DA inhibit ON terminals via D2LRs to reduce ETC burst firing thus indirectly suppresses burst sEPSCs in the ETC-driven PGCs and SACs? To test this, we treated OB slices with bath application of (S)-(-)-Sulpiride (100 μM), a selective D2LR antagonist, for 10 min before addition of DA (Figure 2A). In these conditions, DA reduced sEPSC frequency by 46.8 ± 1.9% (*n* = 7) in PGCs and 49.7 ± 1.9% (*n* = 6) in SACs, amplitude by 47.9 ± 3.7% (*n* = 7) in PGCs and 48.3 ± 3.0% (*n* = 6) in SACs (Figures 2A–C) in the presence of (S)-(-)-Sulpiride. Specifically, sEPSC frequency and amplitude in PGCs (*n* = 7) were 76.8 ± 11.9 Hz and 75.1 ± 11.6 pA in (S)-(-)-Sulpiride, 40.3 ± 5.9 Hz [*P* < 0.0001 vs. (S)-(-)-Sulpiride] and 39.0 ± 6.8 pA [*P* < 0.0001 vs. (S)-(-)-Sulpiride] in (S)-(-)-Sulpiride+DA, and 77.4 ± 12.4 Hz [*P* = 1 vs. (S)-(-)-Sulpiride] and 74.0 ± 11.7 pA [*P* = 1 vs. (S)-(-)-Sulpiride] after washout DA, respectively (Figures 2D,E). Similar results were observed in SACs (*n* = 6) with average frequency and amplitude of 68.2 ± 11.4 Hz and 65.7 ± 12.1 pA in (S)-(-)-Sulpiride, 34.2 ± 6.0 Hz [*P* < 0.0001 vs. (S)-(-)-Sulpiride] and 34.3 ± 6.7 pA [*P* < 0.0001 vs. (S)-(-)-Sulpiride] in the addition of DA, and 67.1 ± 12.0 Hz [*P* = 1 vs. (S)-(-)-Sulpiride] and 64.3 ± 11.8 pA [*P* = 1 vs. (S)-(-)-Sulpiride] after DA washout (Figures 2D,E). These findings suggest that D2LRs do not mediate presynaptic inhibition of glutamate release from ON terminals to ETCs to PGCs and SACs.

Then we examined DA effects on sEPSCs in PGCs and SACs in the presence of the selective D1LR antagonist SKF83566 (10 μM). However, in these conditions, DA altered neither frequency nor amplitude of sEPSCs in either population of glomerular interneurons (Figures 2F–J), implying that the DA suppression of sEPSCs in both PGCs and SACs is mediated by D1LRs.

Altogether, our results support that DA reduces sEPSCs in the ETC-driven PGCs and SACs by activation of D1LRs but this action is not due to reduced ETC activities, which result from activation of D2LRs on ON terminals to reduce glutamate release.

### DA Enhances Spontaneous IPSCs in Glomerular Neurons

**FIGURE 3:**
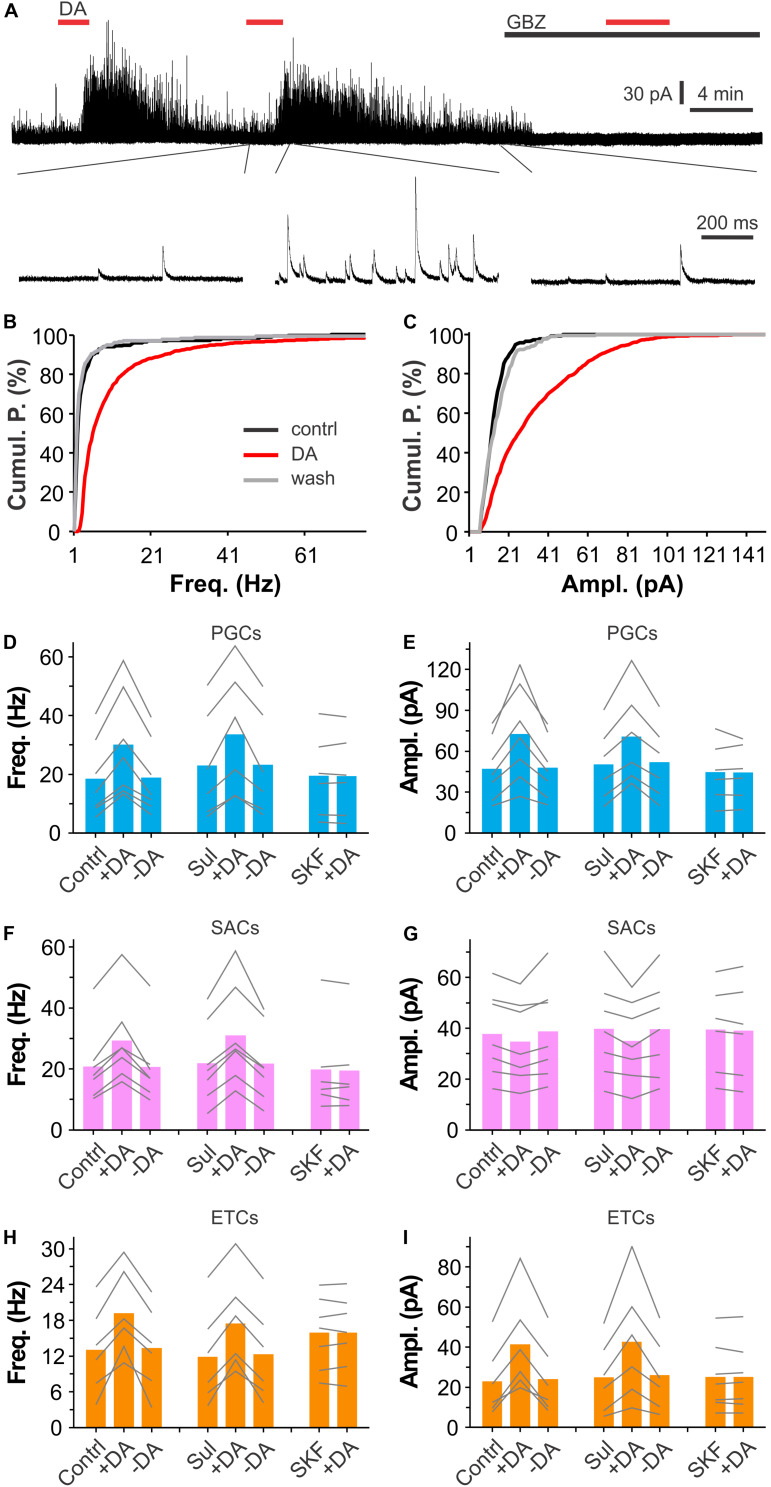
DA enhances synaptic inhibition of glomerular neurons. **(A)** A typical trace (top) showing that bath-applied two doses of DA (20 μM) reversibly and reproducibly enhances spontaneous inhibitory postsynaptic currents (sIPSCs) in a PGC voltage clamped at 0 mV in the presence of NBQX and APV. Note that sIPSCs are completely eliminated by bath-application of the selective GABA_A_ receptor blocker gabazine (10 μM). Bottom traces are blown up from the top traces. **(B,C)** Graphs showing cumulative probability of frequency **(B)** or amplitude **(C)** of sIPSCs presented in A before (control) or during (DA) DA application, or after DA washout (wash). **(D–I)** Pooled data showing that DA significantly enhances sIPSC frequency in PGCs **(D)**, SACs **(F)**, or ETCs **(H)**, and amplitude in both PGCs **(E)** and ETCs **(I)** but not in SACs **(G)** in either control (contrl, *n* = 7 PGCs, 7 SACs, 6 ETCs) or in the presence of 100 μM (S)-(-)-Sulpiride (Sul, *n* = 6 PGCs, 7 SACs, 6 ETCs) while DA has effect on neither frequency nor amplitude of sIPSCs in all three populations of glomerular neurons in the presence of 10 μM SKF83566 (SKF, *n* = 6 PGCs, 6 SACs, 7 ETCs).

Since activation of D1LRs on ETCs produces an excitatory effect (Liu et al., 2013) whereas ON terminals are devoid of D1LRs (Levey et al., 1993; Coronas et al., 1997; Beaulieu and Gainetdinov, 2011), we hypothesized that the DA via D1LRs directly excites PGCs and/or SACs to release GABA, which activates GABA_B_ receptors on ETC apical dendrites (Karpuk and Hayar, 2008) to reduce glutamate release onto PGCs and SACs. In this scenario, DA should elevate the glomerular inhibition. To test this, we recorded spontaneous inhibitory postsynaptic currents (sIPSCs) in voltage clamp in all three populations of glomerular neurons including PGCs, SACs and ETCs, which all have apical dendrites or processes confined to the glomerular layers thus could manifest potential DA-enhanced glomerular inhibition. To maximize detection sIPSCs, which have reversal potential around −70 mV based on chloride concentrations of our internal and external solutions, and minimize sEPSCs, which have a reversal potential at 0 mV, we voltage clamped cells at 0 mV in the presence of NBQX (10 μM) and D-APV (50 μM) to block AMPA and NMDA receptors thus minimize circuit influence. In these conditions, bath application of DA (20 μM) reversibly enhanced sIPSCs in PGCs (Figure 3A). When sIPSCs were completely abolished by the selective GABA_A_ receptor blocker gabazine (GBZ, 10 μM, Figure 3A), DA showed no effect on the holding current, suggesting that DA does not affect PGCs *per se*. Further analysis revealed that both frequency and amplitude of sIPSCs in PGCs were elevated by DA (Figures 3B,C). To determine what receptors mediate these actions, either (S)-(-)-Sulpiride (100 μM) or SKF83566 (10 μM) was bath-applied before DA addition in another set of experiments. It turned out that SKF83566 rather than (S)-(-)-Sulpiride completely blocked DA effects on sIPSCs in all tested PGCs (Figures 3D,E), indicating mediation by D1LRs. Specifically, the average frequency and amplitude of sIPSCs in seven PGCs were 18.4 ± 5.0 Hz and 47.1 ± 8.7 pA in ACSF, 30.0 ± 6.8 Hz (*p* < 0.0001 vs. in ACSF) and 72.5 ± 13.4 pA in DA (*p* < 0.0001 vs. in ACSF), and 18.8 ± 4.8 Hz (*p* = 1 vs. in ACSF) and 47.9 ± 8.5 pA (*p* = 1 vs. in ACSF) after DA washout, respectively. In the presence of (S)-(-)-Sulpiride, average frequency and amplitude of sIPSCs in another set of six PGCs were 23.0 ± 7.5 Hz and 50.2 ± 11.0 pA, 33.6 ± 8.7 Hz (*P* < 0.0001 vs. Sul) and 70.7 ± 14.1 pA (*P* < 0.0001 vs. Sul) with addition of DA, 23.2 ± 7.3 Hz (*p* = 1 vs. Sul) and 51.8 ± 11.1 pA (*p* = 1 vs. Sul), respectively. However, with SKF83566 in the bath, average frequency and amplitude of sIPSCs in six PGCs were 19.5 ± 5.7 Hz and 44.6 ± 9.0 pA, 19.4 ± 5.6 Hz (*p* = 1 vs. SKF) and 44.4 ± 8.3 pA (*p* = 1 vs. SKF) with addition of DA, respectively. Similar pharmacological results of DA actions on sIPSCs were observed in both SACs and ETCs (Figures 3F–I) except DA did not show significant effect on sIPSC amplitude in SACs (Figure 3G), supporting our prediction that DA increases overall glomerular inhibition.

In sum, our results demonstrate that DA enhances both frequency and amplitude of sIPSCs similarly in both PGCs and ETCs while in SACs DA increased only frequency but not amplitude of sIPSCs. Pharmacological analyses suggest that all these elevating effects in all three populations of glomerular neurons are mediated by D1LRs.

### Glomerular Application of DA Augments sIPSCs in Mitral Cells (MCs)

**FIGURE 4:**
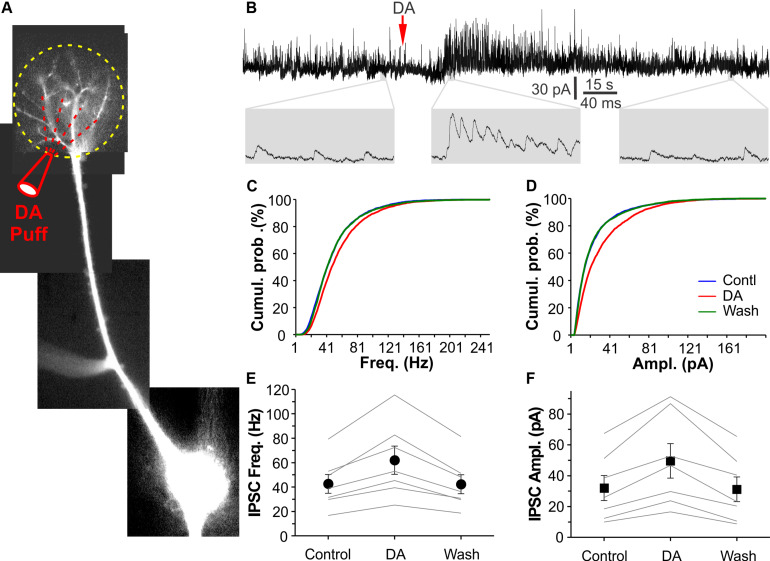
DA elevates glomerular inhibition in MCs. **(A)** Photo graph of a mitral cell filled with Alexa594 via patch clamp pipette in an OB slice. **(B)** The top trace shows that DA (red arrow head) micropuffed to the glomerulus receiving the apical dendrite of the recorded MC as shown in **(A)** potentiates sIPSCs in the MC voltage clamped at 0 mV. Bottom traces are blown up. **(C,D)** Plotting graph showing cumulative probability of sIPSC frequency **(C)** or amplitude **(D)** in control, in the presence of DA, and after DA washout. **(E,F)** Pooled data showing effects of the micropuffed DA on sIPSC frequency **(E)** or amplitude **(F)** in seven MCs.

MCs, the principal OB output neurons, have apical dendrites ramifying in single glomeruli where they receive glomerular inhibition. Thus, it was plausible to predict that DA potentiated glomerular inhibition of MCs. To examine this possibility, we voltage clamped MCs at −0 mV in OB slices to maximize sIPSC detection in the presence of NBQX and APV. MC responses to DA (100 μM) micropuffed (30 psi for 2 ms) to the glomeruli receiving apical dendrites of the recorded cells were recorded as described previously (Shao et al., 2012). Alexa 594 (10 μM) was included in internal solution to visualize the recorded cells and their apical dendrites thus facilitate DA micropuffing (Figure 4A). In these conditions, micropuffing DA reversibly increased both frequency and amplitude of sIPSCs in all tested seven MCs (Figures 4B–F). The average frequency and amplitude of sIPSCs in seven cells were 42.6 ± 7.7 Hz and 32.0 ± 8.7 pA in ACSF (control), 62.0 ± 11.6 Hz (*p* < 0.001 vs. control) and 49.6 ± 11.2 pA (*p* < 0.001 vs. control) in DA, and 42.3 ± 7.8 Hz (*p* = 1 vs. control) and 31.1 ± 8.0 pA (*p* = 1 vs. control) after DA washout, respectively. Collectively, our results demonstrate that DA enhances glomerular inhibition of MCs.

### Dopamine Selectively Excites Short Axon Cells

**FIGURE 5:**
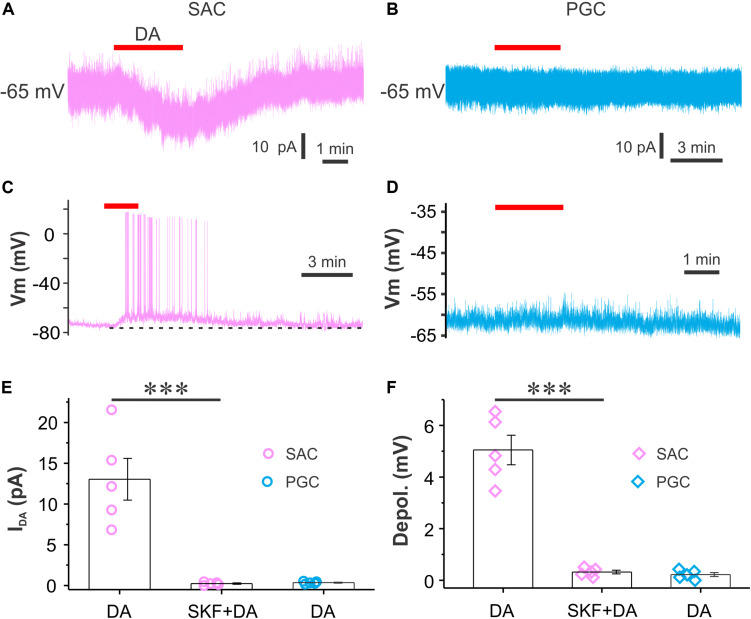
DA directly excites SACs but not PGCs. **(A,B)** Recording traces showing that bath-applied DA produces an inward current in a SAC **(A)** but not in a PGC **(B)** when cells are voltage clamped at –60 mV in the presence of NBQX, APV and GBZ. **(C,D)** Current clamp traces showing that DA depolarizes a SAC **(C)** but not a PGC **(D)**. **(E,F)** Pooled data showing amplitude **(E)** of the DA-induced inward current or depolarization **(F)** in SACs (*n* = 5) but not in PGCs (*n* = 5). ***P < 0.01.

Periglomerular cells and SACs are two major populations of inhibitory glomerular interneurons. To test whether DA activates them to release GABA and increase glomerular inhibition, we did both voltage and current clamp recordings from both SACs and PGCs in the presence of synaptic transmission blockers NBQX (10 μM), APV (50 μM) and GBZ (10 μM) to eliminate circuit influence. SACs responded to bath application of DA with an inward current (Figure 5A) in voltage clamp (*V*_hold_ = −65 mV) or depolarization with spikes in current clamp (Figure 5C) while PGCs did not respond to the same dose of DA (Figures 5B,D). The average amplitude of the DA-induced inward current in five SACs was 13.0 ± 2.6 pA in ACSF and 0.2 ± 0.1 pA in the presence of SKF83566 (*p* < 0.01 vs. in ACSF) while in five PGCs the average amplitude was 0.4 ± 0.1 pA (Figure 5E). In current clamp, the average amplitude of DA-induced depolarization in five SACs was 5.1 ± 0.6 mV in ACSF and 0.3 ± 0.1 mV (*p* < 0.01 vs. in ACSF) in the presence of SKF83566 while there was no depolarization in five PGCs (0.2 ± 0.1 mV) (Figure 5F). Taken together, our results demonstrate that DA selectively excites SACs via D1LRs.

### GABA_B_ Receptors Mediate the DA Suppressive Effects on sEPSCs in Glomerular Interneurons

**FIGURE 6:**
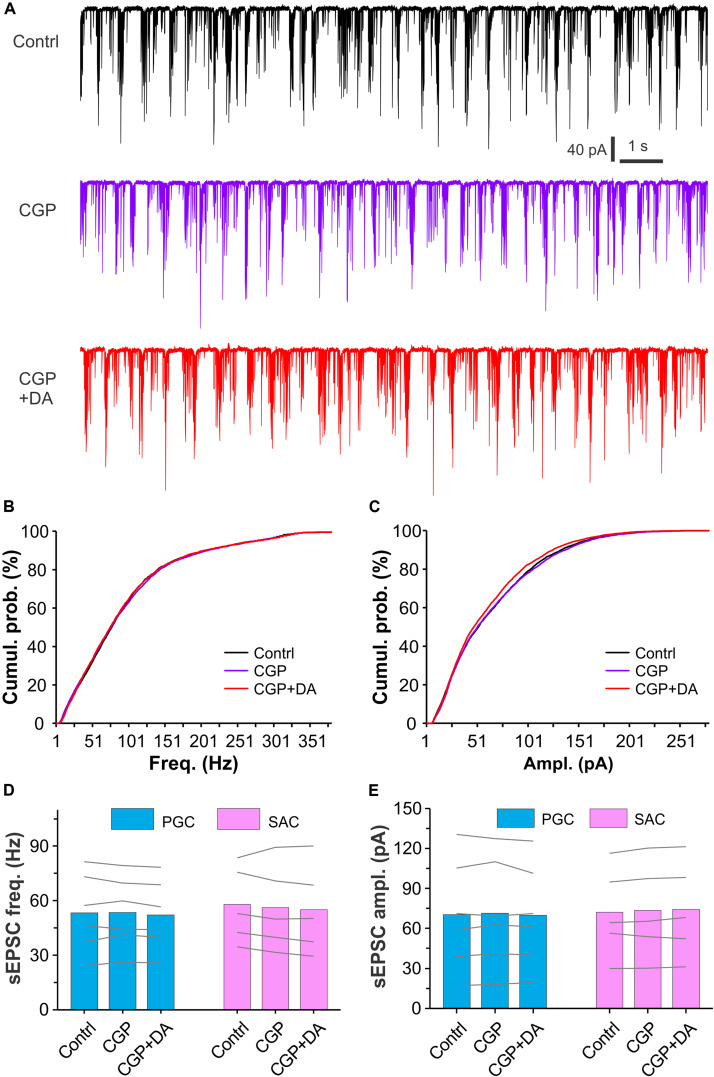
Antagonizing GABA_B_ receptors eliminates DA effects on sEPSCs in glomerular interneurons. **(A)** Typical recording traces showing burst sEPSCs in a PGC before (top), during (middle) bath application of CGP55845 (CGP, 10 μM), a selective GABA_B_ receptor antagonist, or addition of DA (bottom) in the presence of CGP. **(B,C)** Graphs showing cumulative probability of frequency **(B)** or amplitude **(C)** of sEPSCs presented in **(A)**. **(D,E)** Pooled data showing that DA has effect on neither frequency **(D)** nor amplitude **(E)** of sEPSCs in either PGCs (*n* = 6) or SACs (*n* = 5) in the presence of CGP55845.

Dopamine and GABA are coreleased from SACs (Maher and Westbrook, 2008; Borisovska et al., 2013; Liu et al., 2013; McGann, 2013) whereas activation of D2LRs or GABA_B_ receptors produces presynaptic inhibition in many brain areas including the OB (Nickell et al., 1994; Aroniadou-Anderjaska et al., 2000; Ennis et al., 2001; Isaacson and Vitten, 2003; Wachowiak et al., 2005; Beaulieu and Gainetdinov, 2011; McGann, 2013). To test whether activation of GABA_B_ receptors is required for the DA actions we observed, sEPSCs were recorded in both PGCs and SACs and their responses to DA were compared between with and without the selective GABA_B_ receptor antagonist CGP55845 (10 μM) in the bath. As shown in Figures 6A–C, neither CGP55845 nor CGP55845 plus DA showed effect on sEPSCs in a PGC compared to control. Further analysis revealed DA effect on neither frequency nor amplitude of sEPSCs in six PGCs or five SACs (Figures 6D,E), suggesting that activation of GABA_B_ receptors is required for the DA suppressive effects on sEPSCs in PGCs and SACs.

### DA Produces an Excitatory Net Effect on MCs

**FIGURE 7:**
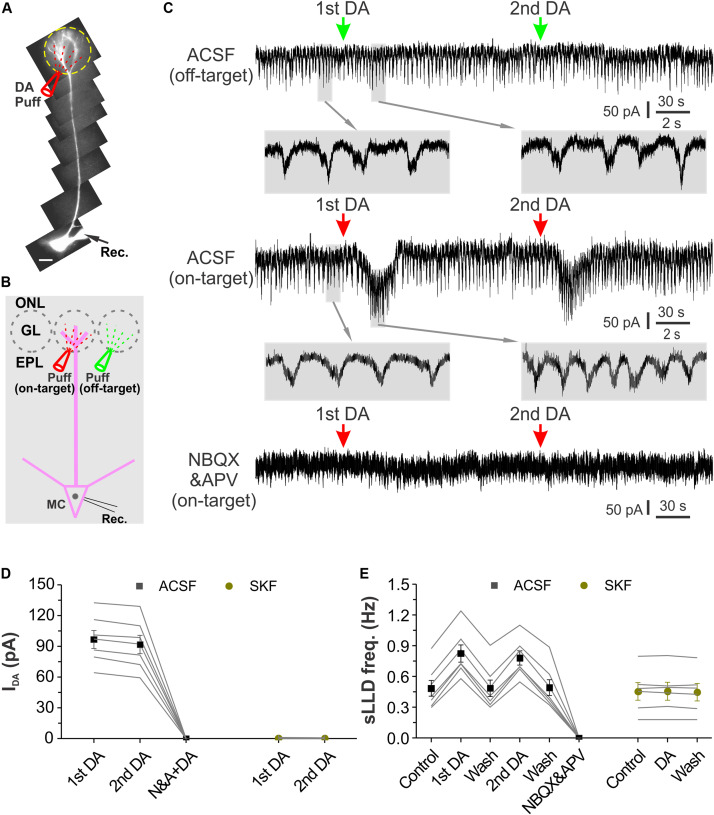
Glomerular application of DA produces an inward current and increases sLLD frequency in MCs. **(A)** Photo graph of a MC filled with Alexa594 via patch clamp pipette in an OB slice. **(B)** Diagram illustration of DA application to the glomerulus (on-target) receiving the apical dendrites of the recorded MC or its adjacent glomerulus (off-target). **(C)** Recording traces showing the rhythmic long-lasting depolarizing (LLD)/inward currents recorded in a MC voltage clamped at –60 mV and their responses to off-target (top), on-target application of DA as shown in **(B)** in control condition (middle) or in the presence of NBQX and APV to block fast glutamate receptors (bottom). Traces at expanded time scale beneath the top and middle traces show changes in LLD current frequency after DA application. **(D)** Graphs of pooled data from two groups of MCs. Data of the first group of MCs (*n* = 7) show the amplitude of the inward currents induced by the first (1st) and second (2nd) dose of DA in ACSF, in the presence of NBQX and APV (N&A). The second group of MCs (*n* = 6) show effects of two doses of DA on-target application on the holding current in the presence of SKF83566, a selective D1LR antagonist. **(E)** Pooled data from two groups of MCs. Data of the first group of MCs (*n* = 7) show the frequency of the spontaneous LLD (sLLD) currents in ACSF (control), addition of the first dose of dopamine (1st DA), washout of the first dose of DA, application of the second dose of DA (2nd DA), washout of the second dose of DA, or in the presence of NBQX and APV. Data of the second group of MCs (*n* = 6) show sLLD frequency in the presence of SKF83566 before (control) or during (DA) DA application, and after DA washout (wash).

External tufted cells provide direct excitatory input to MCs and contribute to long-lasting depolarization (LLD) (De Saint et al., 2009; Gire and Schoppa, 2009). Our previous study showed that DA increases ETC spontaneous spike burst frequency (Liu et al., 2013), indicating that DA increase LLD frequency. However, our present results showed that DA enhanced glomerular inhibition at least partially due to its selective activation of SACs, indicating that DA inhibits MC output. What will be the DA net effects on MC output with integration of these two directionally opposite actions? To answer this question, we first recorded MCs in voltage clamp with a holding potential at −60 mV and observed their responses to glomerular application of DA as shown by Figures 7A,B. Alexa 594 (10 μM) was included in the patch pipette to visualize the recorded MC somata and their apical dendrites. In these conditions, micropuffing DA (100 μM, 2 ms) to glomeruli (off-target) next to the ones receiving the apical dendrites of the recorded cells did not evoke alteration in the spontaneous LLD (sLLD, Figure 7C top traces). However, when the puffing electrode was moved to target the glomerulus innervated by the apical dendrite of the recorded cell (on-target), DA reversibly induced an inward current in MCs (Figure 7C middle traces). The frequency of the spontaneous LLD superimposed on the inward current was increased compared to that before DA application (Figure 7C middle blown-up traces). After bath application of the glutamatergic receptor blockers NBQX and APV, sLLDs disappeared and glomerular application of DA showed no effect on the holding current (Figure 7C bottom trace), indicating that DA does not affect MC intrinsic properties and the DA-induced inward current is mediated by glutamate from ETCs. Consistently, further experiments showed that DA effects on MCs were completely blocked by SKF83566 (Figures 7D,E), indicating that they are due to activation of D1LRs in ETCs (Liu et al., 2013). In seven MCs, the average amplitude of the first and second dose of DA-induced inward current was 96.8 ± 8.7 pA and 91.9 ± 8.9 pA, respectively (Figure 7D). The sLLD frequency in seven MCs was increased from 0.48 ± 0.08 Hz in control to 0.83 ± 0.08 Hz by the first dose of DA but resumed to 0.49 ± 0.08 Hz after DA washout. The second dose of DA elevated this frequency to 0.78 ± 0.07 Hz (*p* < 0.05 compared to washout of first dose of DA) followed by a recovery (0.49 ± 0.08 Hz). In sum, these voltage clamp data showed that glomerular application of DA produced an excitatory net effect on MCs via D1LRs.

**FIGURE 8:**
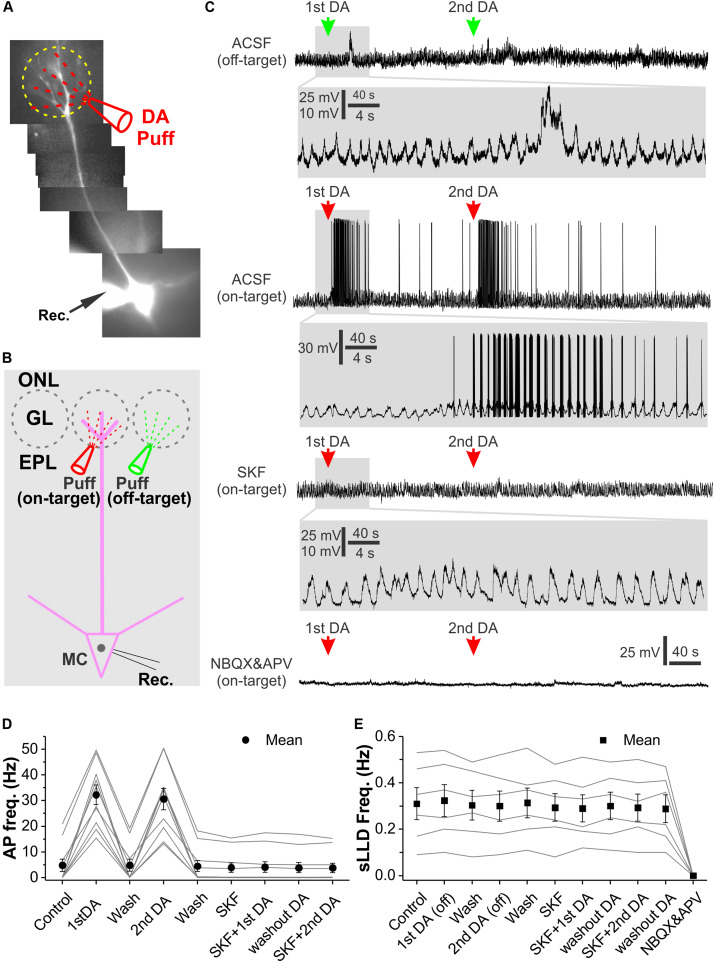
Glomerular application of DA elevates OB output from MCs. **(A)** Photo graph of a MC filled with Alexa594 via patch clamp pipette in an OB slice. **(B)** Diagram illustration of DA application to the glomerulus (on-target) receiving the apical dendrites of the recorded MC or its adjacent glomerulus (off-target). **(C)** Recording traces showing the spontaneous rhythmic long-lasting depolarization (LLD) recorded in a MC in current clamp and their responses to off-target (top) or on-target application of DA as shown in **(B)** in ACSF, in the presence of SKF83566 to block D1LRs (middle) or NBQX and APV to block fast glutamate receptors (bottom). sLLDs are completely eliminated by NBQX and APV. Blown-up traces show DA effects on sLLD frequency or spike firing in each condition. **(D)** Graphs of pooled data from 10 MCs showing effects of two doses of on-target DA on action potential (AP) frequency in ACSF or in the presence of SKF83566. **(E)** Graphs of pooled data from six MCs showing effects of two doses of off-target DA or two doses of on-target DA on sLLD frequency in the presence of SKF83566 as well as effect of NBQX and APV.

This conclusion led us to predict that DA enhance MC output to downstream centers. To test this, we repeated the prior experiments in current clamp (Figures 8A,B). Again, off-target glomerular application of DA did not evoke significant effect on MC membrane potential or sLLD frequency (Figure 8C top traces and Figure 8E) while on-target DA reversibly and reliably evoked spike responses in MCs (Figure 8C middle traces and Figure 8C). Consistent with voltage clamp results, after treatment of OB slices with SKF83566 for 10 min, on-target DA neither elicited spike response nor affected sLLDs (Figures 8C,E) whereas DA no longer showed any effect in the presence of NBQX and APV to completely block sLLDs (Figure 8C bottom trace). The first dose of DA increased the average instant frequency of spikes in 10 MCs from 4.8 ± 2.4 Hz to 32.2 ± 3.8 Hz (*p* < 0.0001) followed by a complete recovery (4.9 ± 2.4 Hz, *p* = 1 compared to pre-DA) while the second dose of DA reversibly elevated it up to 30.6 ± 4.2 Hz (*p* < 0.0001 compared to first DA washout). These actions were completely blocked by SKF83566 (Figure 8D).

Taken together, our results demonstrate that DA produces an excitatory net effect on MCs and consequently increases OB output to downstream olfactory centers.

## Discussion

**FIGURE 9:**
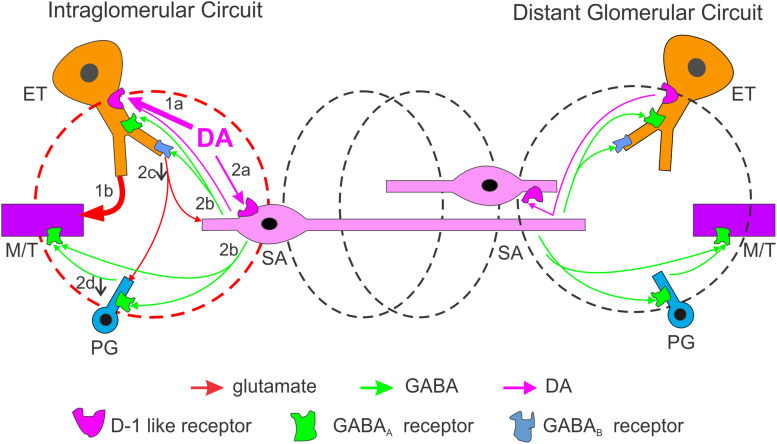
Schematic summarizing DA regulation of OB output through intra- and inter-glomerular circuits. In the activated glomerulus (left, red), the DA net effect on output neurons is excitatory. DA directly excites two glomerular neuron types via D1LRs: the external tufted cells (ETCs, 1a) and short axon cells (SACs, 2a). Excitation of ETCs increases MC output (1b) while excited SACs corelease GABA and DA (2b) which lead to multiple consequences: (1) GABA activates GABA_B_ receptors on ETC apical dendrites to reduce glutamate release and suppress sEPSCs in postsynaptic periglomerular cells (PGCs) and SACs (2c); (2) GABA activates GABA_A_ receptors on PGCs to reduce glomerular inhibition of MCs (disinhibition) leading to further increase in MC output (2d); (3) GABA also inhibits MCs directly, but this inhibition is surpassed by the ETC-MC pathway-derived excitation. In the neighboring or distal glomeruli (black), output neurons are inhibited mainly by GABA-mediated interglomerular action. Thus, DA enhances the system’s sensitivity through a mechanism similar to center-surround inhibition.

We characterized DA actions on both excitatory and inhibitory transmission in the glomerular circuit followed by analysis of the underlying cellular mechanisms and DA impact on OB output with five major findings (Figure 9). First, DA via D1LRs suppresses sEPSCs in the ETC-driven inhibitory glomerular interneurons, contradicting with DA excitatory actions on ETCs. Second, activation of D1LRs potentiates sIPSCs in all glomerular neurons, indicating enhancement of glomerular inhibition. Consistently, glomerular application of DA elevates sIPSCs in MCs. Third, the depression of sEPSCs in inhibitory glomerular interneurons is blocked by inhibition of GABA_B_ receptors, implying that DA increases GABA release from inhibitory glomerular interneurons to inhibit glutamate release from ETC apical dendrites. Fourth, DA selectively excites the GABAergic SACs but not PGCs via D1LRs. Finally, DA produces a net excitatory effect on MC output via D1LRs. This effect is mediated by glutamate, suggesting the DA excitation of ETCs surpasses the enhanced glomerular inhibition thus potentiates the OB output to downstream targets.

### DA Regulates the Excitation–Inhibition Balance in the Glomerular Circuit

Our previous study demonstrates that endogenous DA released from SACs or exogenous DA enhances ETC output by elevating the hyperpolarization-activated cation current (*I*_h_) (Liu et al., 2013). ETCs do not have lateral dendrites and their sole apical dendrites ramify in individual glomeruli (Pinching and Powell, 1971a; Macrides and Schneider, 1982; Hayar et al., 2004b; Antal et al., 2006). Thus, they are generally considered as local excitatory interneurons participating in the operation of glomerular circuit. ETC apical dendrites form glutamatergic dendrodendritic synapses with vast majority of PGCs and SACs (Shao et al., 2009; Kiyokage et al., 2010), and apical dendrites of MTCs (De Saint et al., 2009; Gire and Schoppa, 2009) so that the overall excitation level at the glomerular level has been thought to be controlled or coordinated by ETC activities (De Saint et al., 2009). Subsequent studies with optogenetic approaches did not reveal endogenous DA actions on other postsynaptic targets of SACs including MTCs and PGCs (Banerjee et al., 2015; Liu et al., 2016; Shao et al., 2019), suggesting that DA functions as a neurotransmitter to mediate wiring transmission only from SACs to ETCs. In this context, by activating ETCs DA should increase synaptic excitation across all neuron populations in the glomerular layer. However, DA may alternatively function as a neuromodulator to mediate volume transmission given its long-lasting small quantal release from SACs (Cragg et al., 2001; Borisovska et al., 2013). In this scenario, actions of endogenous DA released from SACs may alter synaptic activities at dendritic terminals without detectable changes in membrane current or potential at cell somata where recordings are normally made from with conventional electrophysiological approaches. In other words, DA could act on neurons other than ETCs to subtly alter the synaptic excitation–inhibition balance among distinct neuron populations. Consistently, findings of the present study support this prediction, i.e., exogenous DA excites only MTCs but not the ETC-driven inhibitory glomerular interneurons PGCs and SACs, in which DA via D1LRs decreases spontaneous excitatory synaptic activities instead. Our further analysis supports that the excitatory actions of DA on MTCs are not due to direct activation of MTCs but instead are through ETC intermediation because they are eliminated by blocking the glutamatergic receptors AMPA and NMDA receptors, which mediate the transmission from ETCs to MTCs (De Saint et al., 2009; Gire and Schoppa, 2009). Thus, DA differentiates the ETC-driven MTCs from ETC-driven PGCs and SACs by resetting the ETC-derived excitatory synaptic input to postsynaptic targets. These findings are consistent with previous studies showing that:(1) DA activates ETCs (Liu et al., 2013) which provide excitatory feedforward to MTCs (De Saint et al., 2009; Gire and Schoppa, 2009); (2) D1LRs are broadly expressed in the OB including the glomerular layer (Coronas et al., 1997; Beaulieu and Gainetdinov, 2011). Therefore, the overall impact of DA actions is to enhance the OB output by enhancing the ETC-intermediated excitatory feedforward to MTCs and weakening ETC-driven excitatory synaptic input to the local GABAergic interneurons PGCs and SACs, which provide inhibitory feedforward to MTCs.

### Cellular Mechanisms Underlying DA Actions

Our mechanistic analysis revealed that the DA-induced suppression of excitatory synaptic activities in ETC-driven PGCs and SACs requires activation of GABA_B_ receptors. Activation of GABA_B_ receptors on OSN axon terminals inhibits glutamate releases (Nickell et al., 1994; Aroniadou-Anderjaska et al., 2000; Wachowiak et al., 2005; McGann, 2013) whereas GABA_B_ receptors are also present in OB glomerular neurons (Fritschy et al., 1999; Panzanelli et al., 2004). Consistent with previous work showing that GABA_B_ receptors inhibit dendrodendritic transmission from MTCs to granule cells in the OB (Isaacson and Vitten, 2003), our finding suggests that GABA released from local interneurons in response to DA activates GABA_B_ receptors on ETC apical dendrites to reduce glutamate release causing suppression of excitatory synaptic activities in postsynaptic PGCs and SACs. This interpretation is supported by our findings that DA via D1LRs augments spontaneous inhibitory synaptic activities in all glomerular neurons and MTCs, suggesting DA increases GABA release from inhibitory interneurons in the glomerular layer. Interestingly, DA selectively excites or depolarizes SACs and increases their spontaneous firing frequency without detectable effect on PGCs. Taken together, these findings lead us to conclude that DA excites SACs, which release GABA to activate GABA_B_ receptors on ETC apical dendrites and reduce glutamate release causing suppression of spontaneous excitatory synaptic activities in PGCs and SACs (Figure 9). Recent studies showed that another type of inhibitory neurons termed deep short axon cells (dSACs) have processes extending into the glomerular layer (Eyre et al., 2008; Burton et al., 2017). Future work needs to examine DA effect on dSACs to see if this population of interneuron are also subject to DAergic modulation.

### Functional Implications

Similar to other brain regions like the striatum, activation of D1LR or D2LRs produces opposite effects on olfactory processing (Yue et al., 2004). In contrast to activation of D2LRs that generally produces inhibitory effects on odor discrimination and transmission of the first synapse from OSNs to postsynaptic targets in the OB (Nickell et al., 1991; Aroniadou-Anderjaska et al., 2000; Ennis et al., 2001; Yue et al., 2004; Tillerson et al., 2006; Escanilla et al., 2009; McGann, 2013), activation of D1LRs improves odor detection and discrimination (Doty et al., 1998; Yue et al., 2004). Consistently, our findings in the present study show that overall net effect of DA on OB output is the D1LR-mediated excitation, which depolarizes the output neurons MTCs to bring their resting membrane potential closer to the threshold of action potential generation, meanwhile, activation of D1LRs selectively excites SACs. Since SACs have long processes connecting multiple glomeruli (Kosaka and Kosaka, 2008; Kiyokage et al., 2010), DA activation of SACs is speculated to extend inhibition to ETCs (Liu et al., 2013; Whitesell et al., 2013) and output neurons (Banerjee et al., 2015; Liu et al., 2016) in neighboring or distal glomeruli. Thus, we propose that activation of D1LRs is to produce a center-surrounding effect thus enhances OB output from the activated glomeruli to facilitate odor detection and discrimination. Further work by knocking out D1LRs or chemogenetic inhibition of D1LRs in the OB would provide insight into this speculation.

## Data Availability Statement

All datasets generated for this study are included in the manuscript.

## Ethics Statement

The animal study was reviewed and approved by Howard University Animal Care and Use Committees.

## Author Contributions

SL planned and performed the experiments, analyzed the data, performed the statistical analysis, interpreted the results, wrote the manuscript, and approved the content.

## Conflict of Interest

The author declares that the research was conducted in the absence of any commercial or financial relationships that could be construed as a potential conflict of interest.

## Footnotes

1 https://hlab.stanford.edu/wdetecta.php
